# A unique case of miliary pulmonary tuberculosis induced by bacillus Calmette-Guérin intravesical instillation with COVID-19 superinfection

**DOI:** 10.4102/sajr.v25i1.2122

**Published:** 2021-06-17

**Authors:** Nicolò Brandi, Laura Bartalena, Cristina Mosconi, Rita Golfieri

**Affiliations:** 1 Department of Radiology, IRCCS University Hospital of Bologna, Bologna, Italy

**Keywords:** tuberculosis, BCG, COVID-19, SARS-CoV-2, CT

## Abstract

Intravesical instillation of Bacillus Calmette-Guérin (BCG) is used as an adjuvant treatment of bladder cancer. Systemic BCG infection occurs in less than 1% of cases, and pulmonary involvement is even rarer (0.3% – 0.7%), with a favourable prognosis. A 78-year-old male developed miliary tuberculosis (TB) secondary to intravesical BCG treatment and subsequent coronavirus disease 2019 (COVID-19) superinfection that led to patient death. High awareness amongst clinicians is needed to proceed with immediate appropriate therapy in these patients, especially during the COVID-19 pandemic

## Introduction

Bacillus Calmette-Guérin (BCG) is a live, attenuated strain of *Mycobacterium bovis,* which is widely used as adjuvant treatment for bladder cancer. Intravesical instillations of BCG are generally well tolerated by patients, with some minor and self-limiting side effects. Systemic BCG infection is reported in less than 1% of cases, and pulmonary involvement is even rarer (0.3% – 0.7%). Pulmonary BCG infection may present as interstitial pneumonitis or miliary dissemination, and its prognosis is generally favourable, with complete resolution after anti-tubercular treatment.^1^

The ongoing coronavirus disease 2019 (COVID-19) pandemic, which started in December 2019 in China, has overwhelmed healthcare systems globally and proved to be a new menace because of its devastating effect on lung function, especially in the more fragile patient.^2^

This report describes the first case of miliary tuberculosis (TB) secondary to intravesical treatment with BCG with sudden worsening of respiratory function caused by COVID-19 superinfection.

## Case presentation

A 78-year-old male with a history of high-grade pTa bladder cancer, treated by transurethral resection and two intravesical instillations of BCG, was admitted to the Emergency Department because of the onset of fever (38 °C), cough, arthralgia, dysuria and pollakiuria. Symptoms started after the last instillation of BCG 10 days earlier. The patient had been empirically treated with co-trimoxazole prescribed by his primary care physician without improvement. Medical history included type 2 diabetes mellitus (on therapy with oral metformin), abdominal aortic aneurysm and chronic obstructive pulmonary disease.

Auscultation revealed generalised decreased vesicular breathing sounds. Blood tests showed increased levels of ferritin (1390 ng/mL), C-reactive protein (8.29 mg/dL) and transaminases (ALT 85 U/L and AST 86 U/L) with normal urinalysis. The severe acute respiratory syndrome coronavirus 2 (SARS-CoV-2) reverse transcription polymerase chain reaction (RT-PCR) test was found to be negative.

**FIGURE 1:**
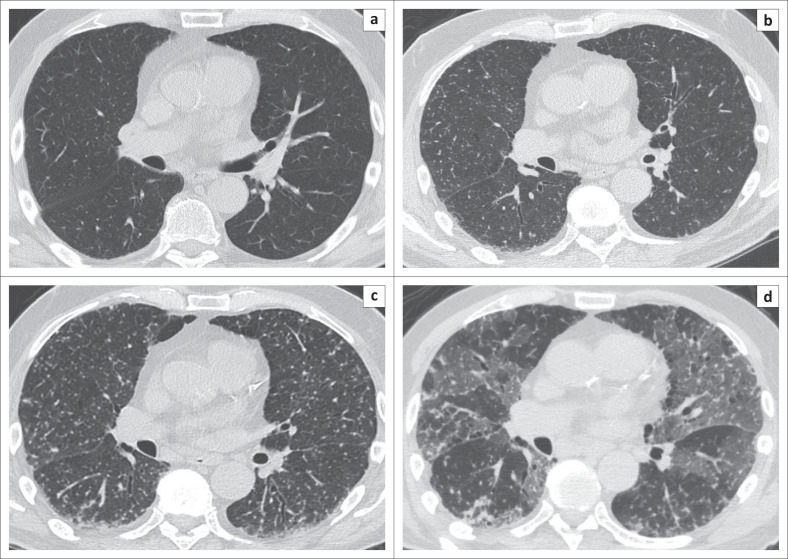
A normal CT of the chest performed before the patient’s access to the Emergency Department (a). A CT scan of the same patient performed 10 days after the last Bacillus Calmette-Guérin instillation, showing 1 mm – 2 mm diameter nodules disseminated throughout the lung and distributed randomly with respect to the lobular structures in a miliary pattern (b). After two weeks of antibiotic therapy, CT imaging revealed a numerical and dimensional increase in the multiple micronodules (c). Despite the administration of anti-tubercular therapy, the patient’s clinical condition deteriorated again, requiring a new CT scan that revealed the presence of bilateral ground-glass opacities localised mainly in the middle and lower parts of the lungs, partially superimposed on the previously seen multiple lung micronodules, compatible with coronavirus disease 2019 superinfection (d).

Upon suspicion of atypical lung infection, a chest CT scan was requested, the result of which was compared with previously obtained normal chest CT findings of the same patient performed a year before his access to the Emergency Department (Figure 1a). The CT scan demonstrated signs of severe centrilobular and paraseptal emphysema, and innumerable lung micronodules (1 mm – 2 mm in size) distributed in the non-emphysematous lung parenchyma as a random pattern (Figure 1b). Since these radiological findings were compatible with numerous diseases, including miliary tuberculosis, sarcoidosis, pneumoconiosis and hematogenous metastases from primary cancers of the thyroid or kidney,^3^ bronchoscopy with bronchoalveolar lavage (BAL) was performed. The cytological examination of the BAL was negative for malignant cells, and samples were negative for bacterial detection, in particular for acid-alcohol-resistant bacilli (both direct and culture), as well as for fungi and other microorganisms.

Given the persistence of fever and dyspnoea after two weeks, Meropenem was administered in the place of the previous antibiotic, and a radiological re-evaluation was carried out with another chest CT, which demonstrated a numerical and dimensional increase in the multiple micronodules (Figure 1c). The patient successively underwent a repeat bronchoscopy with BAL, documenting a positive TB PCR result this time.

Bacillus Calmette-Guérin-itis with pulmonary localisation was finally diagnosed, and therapy with isoniazid, rifampicin and ethambutol was administered with an improvement in the patient’s clinical condition after a few days.

One week after the start of anti-tubercular therapy, the patient’s clinical condition deteriorated again, with a sudden onset of dyspnoea and respiratory distress. Auscultation revealed bilateral diffuse fine crackles and wheezes. A contrast-enhanced chest CT excluded pulmonary embolism but revealed the presence of diffuse ground-glass opacities localised mainly in the middle and lower zones of the lungs, partially superimposed on the previously seen multiple lung micronodules (Figure 1d). As these radiological findings were suggestive of SARS-CoV-2 infection, RT-PCR was repeated again, and this time tested positive, confirming COVID-19 superinfection. The patient succumbed after two days, following respiratory complications.

## Discussion

Immunotherapy with an attenuated live strain of *M. bovis* is the most effective adjunctive therapy for high-risk superficial bladder cancer, which is even more effective than intravesical chemotherapy.^4^ The intravesical instillation of BCG promotes the local activation and migration of numerous polymorphonuclear cells with the consequent release of inflammatory cytokines, ultimately leading to the death of tumour cells. Due to this inflammatory challenge, minor side effects are commonly reported, including cystitis, haematuria, fever, chills and malaise, usually self-limiting within a few hours or days.^1^

Miliary TB as a manifestation of pulmonary involvement of BCG infection is very rare, and only a very few cases have been reported thus far.^1,5,6,7^ Although its pathogenesis remains incompletely understood, some authors consider pulmonary BCGitis to be a type of hypersensitivity reaction as serological tests and cultures are negative in approximately 60% of cases. However, some case reports support the theory of haematogenous spread from an active mycobacterial infection based on the isolation of mycobacteria or the detection of its genome by PCR, as in this case. The presence of underlying immunosuppression, bladder mucosal damage and other patient characteristics are risk factors for disseminated infection.^4^

Imaging plays a key role in the diagnosis of miliary TB and, as in the present case, CT findings guided the diagnosis, even when microbiological and histopathological evidence of mycobacterial infection were negative.^6^ In this case, a presumptive diagnosis of disseminated BCG infection was justified by the clinical presentation consistent with active TB, associated with radiological findings consistent with miliary dissemination of Mycobacterium tuberculosis and high ferritin levels, commonly reported amongst pulmonary TB patients^7^; finally, the evidence of mycobacterial genome by PCR confirmed the diagnosis.

In disseminated BCG infection, the prognosis is generally favourable, and the combination of isoniazid, rifampicin and ethambutol is suggested, with the addition of steroids in severe cases. In this patient however, the sudden superinfection with COVID-19 resulted in a rapid worsening of respiratory function and, eventually, death. A recent review^2^ suggested that tuberculosis and a previous history of tuberculosis seem to be related to an increased susceptibility to COVID-19, as well as worsening of the infection prognosis possibly because of a synergism between the two pathogens. This could explain the poor prognosis of our patient who, moreover, had already two important co-morbidities (i.e. type 2 diabetes mellitus and chronic obstructive pulmonary disease).

## Conclusion

Miliary TB is a very rare complication of intravesical BCG instillation, with unclear aetiopathogenesis, and thus, a high level of suspicion is required for early diagnosis. Chest CT imaging is essential for the diagnosis of this complication because of the high rate of negative microbiological isolations. Finally, high awareness amongst clinicians is necessary to commence appropriate therapy in these patients immediately, especially in the era of the COVID-19 pandemic, as they already present with compromised lung function.
